# Supplementary material for: Spatiotemporal H2O2 flashes coordinate actin cytoskeletal remodeling and regulate cell migration and wound healing
Source: Nat Commun. 2025 Jul 25;16:6868. doi: 10.1038/s41467-025-62272-1 (PMC12297405; doi:10.1038/s41467-025-62272-1)
Supplement: Supplementary file 1 — Supplementary Information [file 41467_2025_62272_MOESM1_ESM.pdf]

Supplementary Information

Spatiotemporal H_2O_2 flashes coordinate actin cytoskeletal remodeling and regulate cell migration and wound healing

Maurice O'Mara, Suisheng Zhang, Ulla G Knaus

Conway Institute, School of Medicine, University College Dublin, Dublin, Ireland

Corresponding author: ulla.knaus@ucd.ie

Conway Institute, School of Medicine, University College Dublin, Dublin, Ireland

Includes: **Supplementary Figures 1-6**

Supplementary Figures

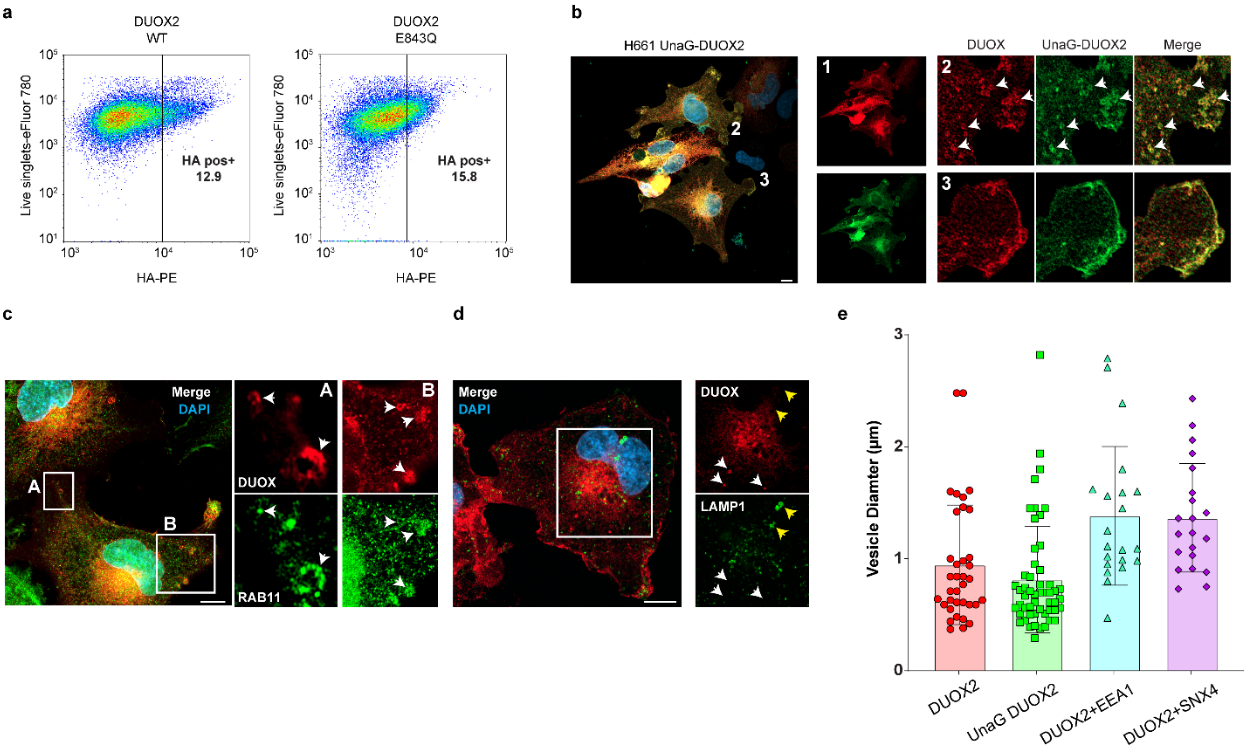

Supplementary Fig. 1: Characterization of DUOX2 vesicular trafficking

a, Flow cytometry analysis of DUOX2 (HA) cell surface localization in H661 DUOXA2 cells transiently transfected with DUOX2 WT or DUOX2 E843Q. Cells were gated for live/dead on the y-axis and HA-PE fluorescence on the x-axis, representative graphs from n=3 experiments **b**, Colocalization of UnaG-DUOX2 (green fluorescence) with anti-DUOX staining (red) in fixed cells, co-stained with DAPI for nuclei (blue), inserts show 1: split channels, 2: vesicle colocalization, 3: plasma membrane colocalization. **c**, Immunofluorescence staining of DUOX2 (red) and RAB11 (green) vesicles in H661 DUOX2 WT cells, inserts show digital magnification of areas denoted by white boxes, white arrows indicate vesicles. **d**, Immunofluorescence staining of DUOX2 (red) and LAMP1 (green) vesicles in H661 DUOX2 WT cells, inserts show digital magnification of area denoted by white box, white arrows indicate DUOX2 positive/LAMP1 negative vesicles, yellow arrows indicate LAMP1 positive/DUOX2 negative vesicles. **e**, Quantification of intracellular DUOX2 containing vesicle diameters, n=each point represents one vesicle diameter, bars show mean diameter ± SD. Scale bars 10µm, microscopy analyses representative of n=5.

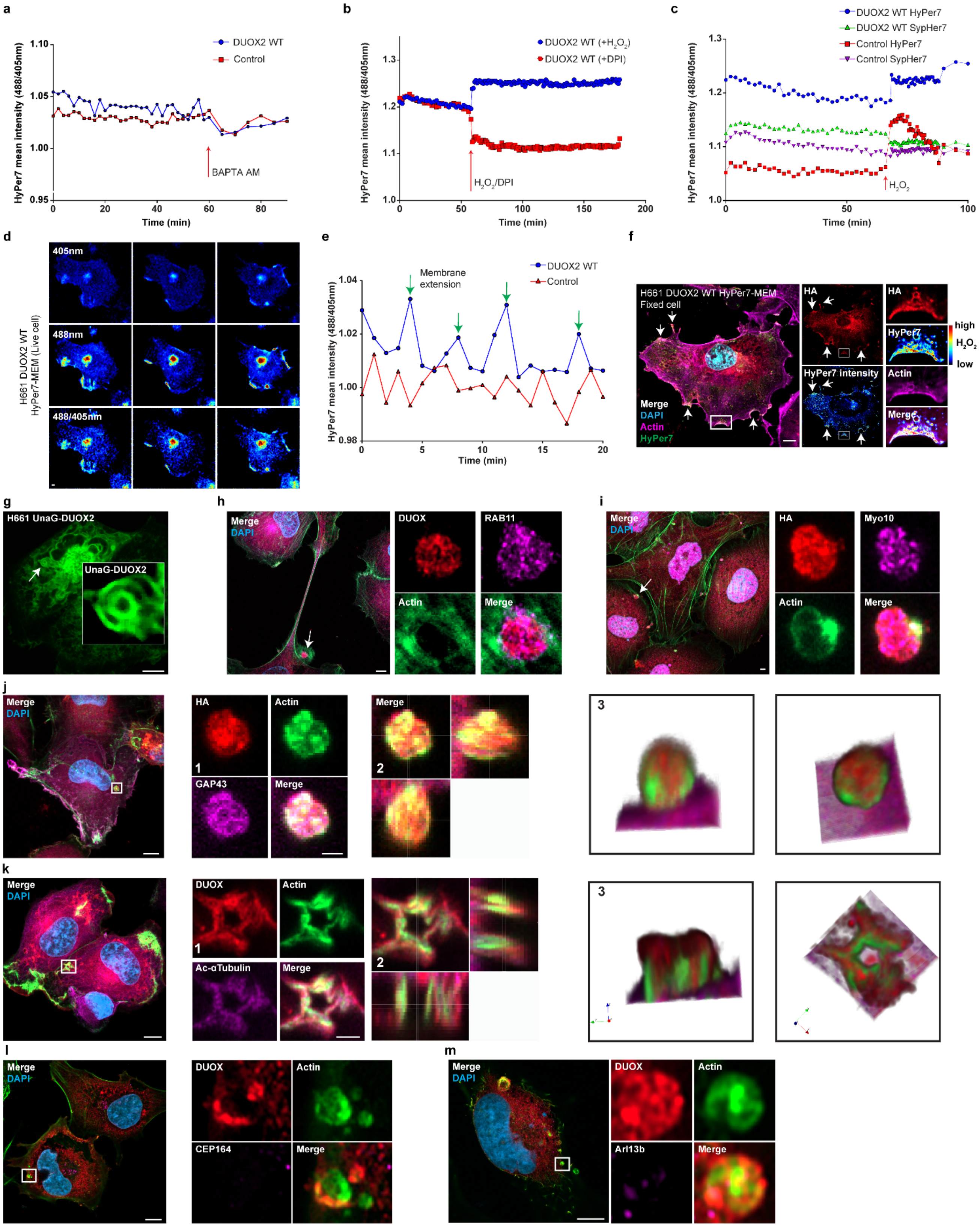

a
HyPer7 mean intensity (488/405nm)
Time (min)
DUOX2 WT
Control
BAPTA AM
b
DUOX2 WT (+H_2O_2)
DUOX2 WT (+DPI)
H_2O_2/DPI
c
DUOX2 WT HyPer7
DUOX2 WT SypHer7
Control HyPer7
Control SypHer7
H_2O_2
d
H661 DUOX2 WT HyPer7-MEM (Live cell)
405nm
488nm
488/405nm
e
Membrane extension
DUOX2 WT
Control
f
H661 DUOX2 WT HyPer7-MEM Fixed cell
Merge
DAPI
Actin
HyPer7
HA
HyPer7 intensity
high
H_2O_2
low
g
H661 UnaG-DUOX2
UnaG-DUOX2
h
DUOX
RAB11
i
Myo10
j
GAP43
k
Ac-αTubulin
l
CEP164
m
Arl13b

Supplementary Fig. 2: DUOX2 activity induces tunnelling nanotube (TNT) formation

a-**c**, Mean HyPer7-MEM 488/405nm ratio signal in H661 cell lines, each point represents the mean HyPer7 ratio from 3-6 cells measured per field of view, 3 fields of view per condition. **a**, HyPer7-MEM expressing DUOX2 WT and control H661 cells recorded for 60min, followed by treatment with BAPTA-AM and recorded for a further 30min. **b**, H661-DUOX2 WT cells expressing HyPer7-MEM were treated with H_2O_2 or diphenyleneiodonium chloride (DPI) at 60min and recorded for 2h. **c**, H661 DUOX2 WT and control cells were transfected with HyPer7-MEM or SypHer7-MEM. Cells were treated with H_2O_2 at 60min and recorded for 40min. **d**, Live cell image of H661 DUOX2 WT HyPer7-MEM cells, showing HyPer7 405nm signal (top) and signal peak colocalization of the 488nm channel (middle) and the 488/405nm ratio (bottom) (Supplementary Movie 3). **e**, Mean HyPer7-MEM 488/405nm ratio signals in membrane protrusions of DUOX2 WT (blue) and Control (red) H661 cell lines; green arrows indicate H_2O_2 generation during membrane extension. **f**, Fixed cell staining of H661 DUOX2 WT HyPer7-MEM cells showing colocalization of DUOX2 (HA, red) and 488nm HyPer7 signal (pixel intensity heatmap); white arrows indicate areas of colocalization, inserts show split channel digital zoom of area in white box. **g**, Live cell image of H661 cells expressing UnaG-DUOX2 (green) depicting a double ring apical protrusion, insert shows digital zoom of area at white arrow. **h**, **i**, Localization of **h**, RAB11 (purple) and **i**, Myo10 (purple) in DUOX2 (HA, red) positive protrusions in H661 DUOX2 WT cells; inserts show split channel digital zoom of areas at white arrows. **j**, GAP43 (purple) localization to DUOX2 (HA, red) expressing apical protrusion in H661 DUOX2 WT cells, inserts 1: split channel digital zoom of area denoted by white box, 2: merged channel x/y z-stack projection of apical protrusion in white box, 3: merged channel 3D rendering of z-stack showing apical protrusion in white box. **k**, DUOX2 (red) localization to circular dorsal ruffles (CDR), containing acetylated α-tubulin (purple) in H661 DUOX2 WT cells; inserts 1: split channel digital zoom of CDR denoted by white box, 2: merged channel x/y z-stack projection of CDR in white box, 3: merged channel 3D rendering of z-stack showing CDR in white box. **l**, **m**, Primary cilia staining in H661 DUOX2 WT cells, indicating DUOX2 (red) is not colocalizing with **l**, CEP164 (purple) or **m**, Arl13b (purple). **f**, **h-m**, co-stained with actin (phalloidin; **f**: purple, **h-m**: green) and DAPI nuclear stain (blue). Scale bars 10μm, microscopy analyses representative of n=5.

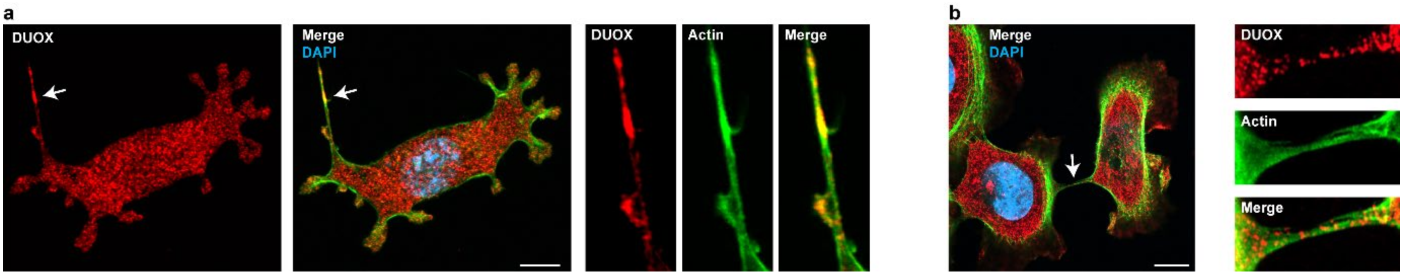

Supplementary Fig. 3: Endogenous DUOX localization in TNTs formed by BxPC3 cells

a, DUOX (red) localization to an extending TNT (white arrow) in BxPC3 DUOX1/2 WT cells; insert show split channel digital zoom of TNT. **b**, DUOX (red) localization within a mature connected TNT (white arrow) between two BxPC3 DUOX1/2 WT cells. **a**, **b**, Co-stained with actin (green) and DAPI (blue). Scale bars 10μm, microscopy analyses representative of n=5.

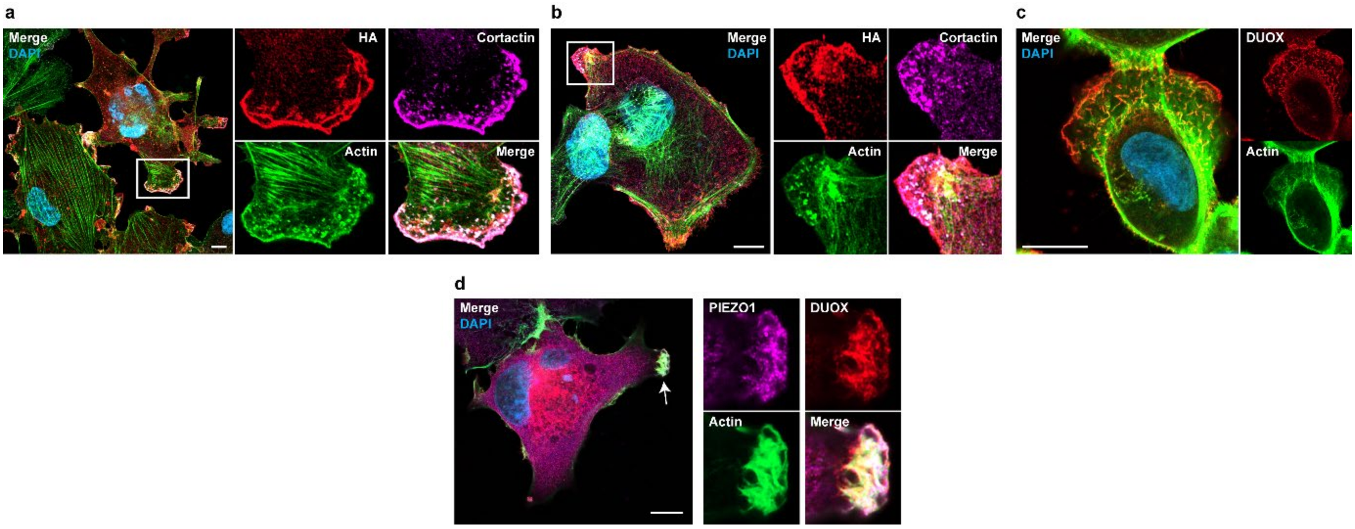

Supplementary Fig. 4: DUOX2 promotes lamellipodia formation

a, **b**, DUOX2 (HA, red) localized to mature (**a**) and extending (**b**) lamellipodia in H661 DUOX2 WT cells, colocalization with cortactin (purple) and actin (green), inserts show split channel digital zoom of areas denoted by white boxes. **c**, DUOX (red) localization to the leading edge of an extending lamellipodia in BxPC3 DUOX1/2 WT cells, co-stained with actin (green). **d**, PIEZO1 (purple) staining in H661 DUOX2 WT cells, colocalized with DUOX2 (red) at a membrane protrusion, co-stained with actin (green). **a-d,** co-stained with DAPI (blue). Scale bars 10µm, microscopy analyses representative of n=5.

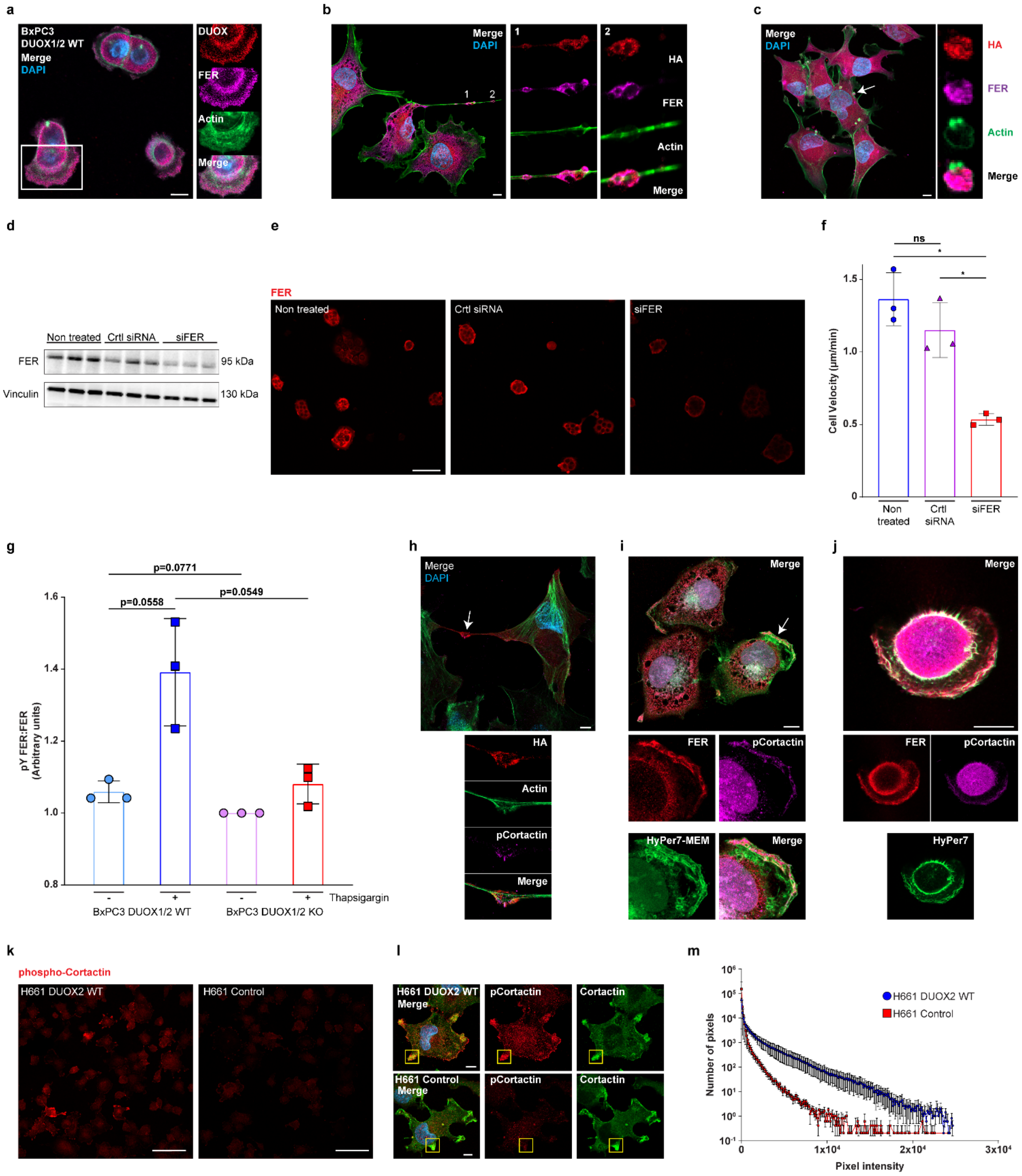

Supplementary Fig. 5: DUOX2/FER/pCortactin colocalization and phosphorylation analyses

a, Colocalization of DUOX (red) and FER (purple) in migrating DUOX1/2 WT BxPC3 cells; insert shows digital zoom of leading edge colocalization. **b,** FER (purple) and DUOX2 (HA, red) colocalization in TNT vesicles in H661 DUOX2 WT cells, inserts show split channel digital zoom of

vesicles at 1 and 2. **c**, Localization of FER (purple) in DUOX2 (HA, red) expressing apical protrusion in H661 DUOX2 WT cells; inserts show split channel digital zoom of area at white arrow. **d**, **e**, Analysis of FER expression by Western Blot (**d**) and confocal microscopy (**e**, red) with and without FER siRNA knockdown. **f**, Single cell migration velocity in non-treated (blue), in negative control siRNA (purple) or FER targeted siRNA pool (red) treated DUOX1/2 WT BxPC3 cells, each point represents the average from 3 independent experiments with 15 cells tracked per experiment, Welch's t-test, **P*<0.05. **g**, Densitometry analysis of pY FER from FER IPs in indicated BxPC3 cells, n= each point represents a single biological replicate, Welch's t-test, p-values displayed on graph. **h**, Phospho-Cortactin (purple) localization to DUOX2 (HA, red) expressing vesicle along a mature connected TNT between H661 DUOX1/2 WT cells, inserts show split channel digital zoom of vesicle at white arrow. **i**, **j**, Plasma membrane colocalization of FER (red) and phospho-Cortactin (purple) in H661 DUOX2 WT HyPer7-MEM (green) cells (**i**) and in BxPC3 DUOX1/2 WT HyPer7-MEM cells (**j**). **k**, Phospho-Cortactin (red) staining in H661 cell lines. **l**, Comparison of both phospho-Cortactin (red) and total Cortactin (green) between DUOX2 WT and Control H661 cell lines. **m**, Quantification of the mean phospho-Cortactin pixel intensity in H661 membrane protrusions, data presented as mean ±SD. **a-c**, **h,** co-stained with actin (green). **a-c, h**, **l**, co-stained with DAPI (blue). **a-c, h-j, l** scale bars 10µm, **e**, **k** scale bars 100µm, microscopy analyses representative of n=5.

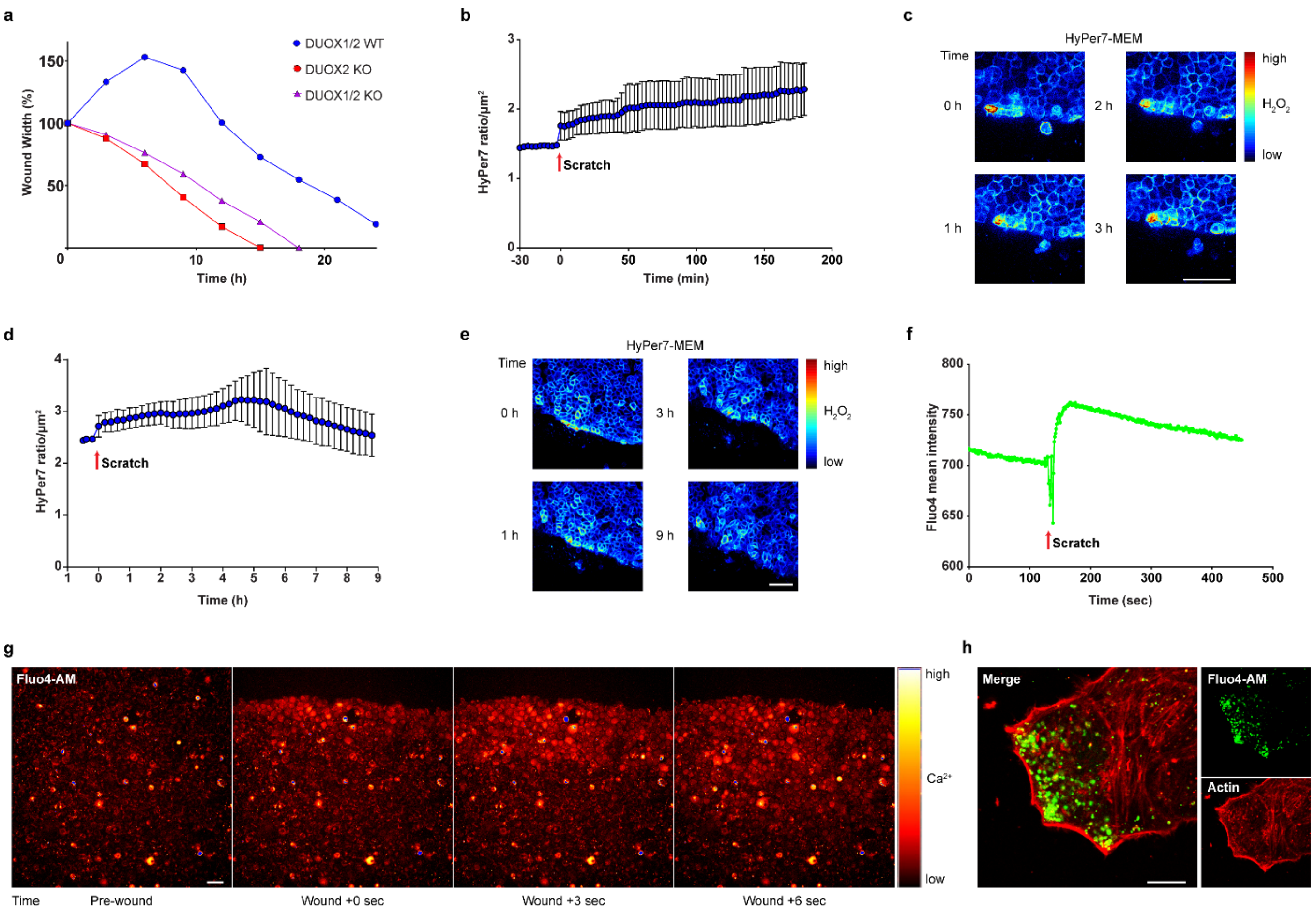

Supplementary Fig.6: DUOX2 generated H_2O_2 is essential for the retraction wave during epithelial wound healing

a, Change in wound width (% of initial wound in scratch assay) in BxPC3 DUOX1/2 WT (blue), DUOX2 KO (red) and DUOX1/2 KO (purple) cell lines. **b-e**, Analysis of HyPer7-MEM oxidation at the wound site of BxPC3 DUOX1/2 WT cells expressing HyPer7-MEM. Average 488/405nm HyPer7 ratios were generated over the initial 3h (**b**) and 9h (**d**) post scratching, both with 30min pre-wound recording. Each point represents the average of 5 independent experiments ±SD, with 2 wells analyzed per experiment. Average HyPer7 ratio was normalized to area of cell coverage (μm^2) at each timepoint. **c**, **e**, Live cell confocal video frames extracted from analysis in **b** and **d**, showing changes in HyPer7 488/405nm ratio at the indicated timepoints, initial 3h (**c**, Supplementary Movie 8) and up to 9h (**e**, Supplementary Movie 8). **f**, Quantification of mean Fluo4-AM signal in BxPC3 DUOX1/2 WT cells during scratch assay; single representative experiment presented. **g**, Frames extracted from scratch assays stained with Fluo4-AM (pseudo colored red heatmap). **h**, Live cell Fluo4-AM positive (green) calcium containing vesicles at the leading edge of a H661 DUOX2 WT cell, co-stained with actin (red). Scale bars **c**, **e**, **g** 100µm, **h** 10µm, microscopy analyses representative of n=5.
