# Supplementary material for: Spatiotemporal H2O2 flashes coordinate actin cytoskeletal remodeling and regulate cell migration and wound healing
Source: Nat Commun. 2025 Jul 25;16:6868. doi: 10.1038/s41467-025-62272-1 (PMC12297405; doi:10.1038/s41467-025-62272-1)
Supplement: Supplementary file 2 — Description of Additional Supplementary Information [file 41467_2025_62272_MOESM2_ESM.pdf]

Description of Additional Supplementary Information

Supplementary Data 1: List of antibodies and cell lines used in this study.

Supplementary Movie 1: Transient membrane and vesicle localization of UnaG-DUOX2

Cell surface and vesicular localization of UnaG-DUOX2 in H661-DUOXA2 cells. Transitions to a higher digital zoom, highlighting UnaG-DUOX2 membrane localization dynamics and internalization. Recorded with confocal imaging at 20 sec per image (frame 5.8 sec, interval 15 sec). Scale bar 10μm.

Supplementary Movie 2: DUOX2 vesicles trafficking along TNT

DUOX2 vesicle transport along a TNT connecting two H661 UnaG-DUOX2 cells. Transitions to higher digital zooms, highlighting vesicle exchange at the point of TNT contact. Recorded with confocal imaging at 20 sec per image (frame 5.8 sec, interval 15 sec). Scale bar 10μm.

Supplementary Movie 3: Colocalization of HyPer7-MEM 488nm and 488/405 ratio membrane peaks

HyPer7-MEM pixel intensity peaks in the 488nm channel (left) match the localization of intensity peaks after generation of 488/405nm ratio (right). H661 DUOX2 WT HyPer7-MEM cells imaged with a spinning disk confocal microscope at 2 min intervals. Scale bar 10μm.

Supplementary Movie 4: H_2O_2 generation in TNT trafficking and extension

HyPer7-MEM signal peaks in vesicles and at the tip of various TNTs in both H661 DUOX2 WT and BxPC3 DUOX1/2 WT cell lines. Transitions through four example TNTs, two in each cell line. Recorded with confocal imaging at 10 sec intervals. HyPer7 488nm channel presented. Scale bar 10μm.

Supplementary Movie 5: DUOX2 localization at the leading edge of extending lamellipodia

Localization of UnaG-DUOX2 during lamellipodia formation in transfected H661 DUOXA2 cells. Transitions to higher digital zoom, highlighting UnaG-DUOX2 localization to the membrane as lamellipodium extends, followed by internalization once extension ceases. Recorded with confocal imaging at 20 sec per image (frame 5.8 sec, interval 15 sec). Scale bar 10μm.

Supplementary Movie 6: H_2O_2 generation at the leading edge of migrating cells

H_2O_2 dynamics during single cell migration in BxPC3 DUOX1/2 WT HyPer7-MEM cells, highlighting high HyPer7 signal intensity at both the leading and trailing edge of migrating cells. Recorded with confocal imaging at 10 sec intervals. HyPer7 488nm channel presented. Scale bar 10µm.

Supplementary Movie 7: Loss of DUOX2 activity alters wound closure dynamics

Difference in wound healing dynamics between BxPC3 DUOX1/2 WT and DUOX1/2 KO cell monolayers after physical scratching. Brightfield microscopy imaged at 30 min intervals. Scale bar 100µm.

Supplementary Movie 8: H_2O_2 generation during wound healing

H_2O_2 generation during the initial 3h post scratching followed by 9h cell front retraction and extension during wound healing in BxPC3 DUOX1/2 WT HyPer7-MEM cells. Imaged with a spinning disk confocal microscope at 30 sec intervals for 3h time series, and 9h at 3 min intervals. HyPer7 488/405nm ratio presented. Scale bar 10µm.

Supplementary Movie 9: **Calcium flux in scratch wound dynamics**

Calcium influx visualized with Fluo4-AM. Starting with two examples of a calcium wave initiated by mechanical scratching of confluent BxPC3 DUOX1/2 WT monolayers at 20X magnification on a spinning disc confocal microscope, scale bar 100µm. Followed by Fluo4-AM increased intensities in BxPC3 DUOX1/2 WT cells preceding cell retraction, and afterwards visualizing calcium flashes at the plasma membrane of H661 DUOX2 WT cells as membrane extension occurs, both imaged at 63X on an Olympus FV3000 microscope. Fluo4-AM intensities are displayed using a pseudo-color red heatmap, giving a clear indication in the changes in bound calcium concentrations. The live actin stain SPY555 FastAct is displayed as white in single cell movies. Scale bar 10µm.

Supplementary Movie 10: DUOX2 deficiency leads to aborted retraction wave upon PIEZO1 stimulation

Wound healing dynamics in BxPC3 DUOX1/2 KO cells treated with PIEZO1 agonist YODA1 (5 µM) during scratch assay. Brightfield microscopy imaged at 30 min intervals. Scale bar 100µm.
