# Supplementary material for: Spatiotemporal H2O2 flashes coordinate actin cytoskeletal remodeling and regulate cell migration and wound healing
Source: Nat Commun. 2025 Jul 25;16:6868. doi: 10.1038/s41467-025-62272-1 (PMC12297405; doi:10.1038/s41467-025-62272-1)
Supplement: Supplementary file 14 — Reporting Summary [file 41467_2025_62272_MOESM14_ESM.pdf]

nature portfolio

Corresponding author(s): Ulla G Knaus

Last updated by author(s): 07/07/2025

Reporting Summary

Nature Portfolio wishes to improve the reproducibility of the work that we publish. This form provides structure for consistency and transparency in reporting. For further information on Nature Portfolio policies, see our Editorial Policies and the Editorial Policy Checklist.

Statistics

For all statistical analyses, confirm that the following items are present in the figure legend, table legend, main text, or Methods section.

n/a	Confirmed	
☐	☒	The exact sample size (*n*) for each experimental group/condition, given as a discrete number and unit of measurement
☐	☒	A statement on whether measurements were taken from distinct samples or whether the same sample was measured repeatedly
☐	☒	The statistical test(s) used AND whether they are one- or two-sided<br>*Only common tests should be described solely by name; describe more complex techniques in the Methods section.*
☐	☒	A description of all covariates tested
☒	☐	A description of any assumptions or corrections, such as tests of normality and adjustment for multiple comparisons
☐	☒	A full description of the statistical parameters including central tendency (e.g. means) or other basic estimates (e.g. regression coefficient) AND variation (e.g. standard deviation) or associated estimates of uncertainty (e.g. confidence intervals)
☐	☒	For null hypothesis testing, the test statistic (e.g. *F*, *t*, *r*) with confidence intervals, effect sizes, degrees of freedom and *P* value noted<br>*Give P values as exact values whenever suitable.*
☒	☐	For Bayesian analysis, information on the choice of priors and Markov chain Monte Carlo settings
☒	☐	For hierarchical and complex designs, identification of the appropriate level for tests and full reporting of outcomes
☒	☐	Estimates of effect sizes (e.g. Cohen's *d*, Pearson's *r*), indicating how they were calculated

Our web collection on statistics for biologists contains articles on many of the points above.

Software and code

Policy information about availability of computer code

Data collection	Zeiss ZEN Blue, Olympus Fluoview, QuantStudio™ Real-Time PCR Software v1.3
Data analysis	ICY Bioimage analysis software (Institut Pasteur), Graphpad Prism 8, FiJi, QuantStudio™ Real-Time PCR Software v1.3

For manuscripts utilizing custom algorithms or software that are central to the research but not yet described in published literature, software must be made available to editors and reviewers. We strongly encourage code deposition in a community repository (e.g. GitHub). See the Nature Portfolio guidelines for submitting code & software for further information.

Data

Policy information about availability of data

All manuscripts must include a data availability statement. This statement should provide the following information, where applicable:

- Accession codes, unique identifiers, or web links for publicly available datasets
- A description of any restrictions on data availability
- For clinical datasets or third party data, please ensure that the statement adheres to our policy

All data supporting the results of this study are available in the main text or in Supplementary Information

Research involving human participants, their data, or biological material

Policy information about studies with human participants or human data. See also policy information about sex, gender (identity/presentation), and sexual orientation and race, ethnicity and racism.

Reporting on sex and gender	*Use the terms sex (biological attribute) and gender (shaped by social and cultural circumstances) carefully in order to avoid confusing both terms. Indicate if findings apply to only one sex or gender; describe whether sex and gender were considered in study design; whether sex and/or gender was determined based on self-reporting or assigned and methods used. Provide in the source data disaggregated sex and gender data, where this information has been collected, and if consent has been obtained for sharing of individual-level data; provide overall numbers in this Reporting Summary. Please state if this information has not been collected. Report sex- and gender-based analyses where performed, justify reasons for lack of sex- and gender-based analysis.*
Reporting on race, ethnicity, or other socially relevant groupings	*Please specify the socially constructed or socially relevant categorization variable(s) used in your manuscript and explain why they were used. Please note that such variables should not be used as proxies for other socially constructed/relevant variables (for example, race or ethnicity should not be used as a proxy for socioeconomic status). Provide clear definitions of the relevant terms used, how they were provided (by the participants/respondents, the researchers, or third parties), and the method(s) used to classify people into the different categories (e.g. self-report, census or administrative data, social media data, etc.) Please provide details about how you controlled for confounding variables in your analyses.*
Population characteristics	*Describe the covariate-relevant population characteristics of the human research participants (e.g. age, genotypic information, past and current diagnosis and treatment categories). If you filled out the behavioural & social sciences study design questions and have nothing to add here, write "See above."*
Recruitment	*Describe how participants were recruited. Outline any potential self-selection bias or other biases that may be present and how these are likely to impact results.*
Ethics oversight	*Identify the organization(s) that approved the study protocol.*

Note that full information on the approval of the study protocol must also be provided in the manuscript.

Field-specific reporting

Please select the one below that is the best fit for your research. If you are not sure, read the appropriate sections before making your selection.

☒ Life sciences ☐ Behavioural & social sciences ☐ Ecological, evolutionary & environmental sciences

For a reference copy of the document with all sections, see nature.com/documents/nr-reporting-summary-flat.pdf

Life sciences study design

All studies must disclose on these points even when the disclosure is negative.

Sample size	Statistical tests were not performed to predetermine the sample size. Sample sizes were chosen based on both the standard sample sizes commonly used in the field and our previous experience with these assays, eg. 3 biological replicates for HVA assays, 20-25 cells tracked per migration assay replicates, all sample size details are listed in both the methods and figure legends.
Data exclusions	No data was excluded from the results reported here.
Replication	Multiple, independent experiments were performed to validate the reproducibility of experiments, all replications were reproduced successfully.
Randomization	No randomization was used in the study. Experiments were individually designed to account for any covariates.
Blinding	No blinding was used in this study.

Reporting for specific materials, systems and methods

We require information from authors about some types of materials, experimental systems and methods used in many studies. Here, indicate whether each material, system or method listed is relevant to your study. If you are not sure if a list item applies to your research, read the appropriate section before selecting a response.

Materials & experimental systems

n/a	Involved in the study
	[x] Antibodies
	[x] Eukaryotic cell lines
[x]	Palaeontology and archaeology
[x]	Animals and other organisms
[x]	Clinical data
[x]	Dual use research of concern
[x]	Plants

Methods

n/a	Involved in the study
[x]	ChIP-seq
	[x] Flow cytometry
[x]	MRI-based neuroimaging

Antibodies

Antibodies used

Primary antibodies used: mouse monoclonal anti-Actin (Cytoskeleton, AAN02-S), rabbit polyclonal anti-F-Actin (Sigma Aldrich, A0266) 1:4000, mouse monoclonal anti-acetylated Alpha tubulin (Sigma Aldrich, T7451) 1:100, rabbit monoclonal anti-EEA1 (Cell Signaling Technology 3288S), mouse monoclonal anti-LAMP1 (DSHB H4A3-c), rabbit monoclonal anti-GAP43 (Invitrogen MA5-32256), rabbit monoclonal anti-Beta tubulin (Epitomics, EP1331Y) 1:1000, mouse anti-phosphotyrosine (Upstate, 05-321) 1:500, rabbit anti-Cortactin (Epitomics, EP1922Y) 1:100, rabbit polyclonal anti-phospho Cortactin (Tyr421) (Invitrogen, 44-854G) 1:100, mouse monoclonal anti-FER (Invitrogen, MA5-15357) IF 1:100, IB 1:500), rabbit polyclonal anti-FER (Proteintech, 25287-I-AP) IF 1:100, IP 2µg), mouse monoclonal anti-PIEZO1 (Invitrogen, MA5-32876) 1:100, rabbit polyclonal anti-Myo10 (Invitrogen, PA5-55019) 1:100, mouse anti-RAB11 (BD Transduction, 610656) 1:100, rabbit polyclonal anti-SNX4 (Synaptic Systems 392 003) 1:100, rabbit polyclonal anti-ARL13B (Proteintech, 17711-1-AP) 1:100, rabbit polyclonal anti-CEP164 (Proteintech ,16851765) 1:800, mouse monoclonal anti-HA tag (Covance, MMS-101P) IF 1:100, IB 1:1000, rabbit anti-DUOX (custom rabbit antibody #7936 1 IF 1:100, rabbit anti DUOX2 (custom rabbit antibody #7959 2 IB 1:1000.

Validation

Antibodies used in the study are commercially available, validation of each primary antibody for the species and application is available from manufacturer (statements on manufacturers' website)
Custom rabbit DUOX #7936 and DUOX2 #7959 have previously been validated in our lab, see Pacquelet, S. et al. J Biol Chem 283, and 24649-24658 (2008) and Luxen, S., Belinsky, S.A. & Knaus, U.G. Cancer Res 68, 1037-1045 (2008).

Eukaryotic cell lines

Policy information about cell lines and Sex and Gender in Research

Cell line source(s)

BxPC-3 Human Epithelial Pancreas (ATCC CRL-1687), HEK-293FT Human Epithelial Kidney Embryo (ATCC CRL-3216), NCI-H661 Human Epithelial Lung (ATCC HTB-183)

Authentication

BxPC-3, HEK-293FT and NCI-H661 cell lines were authenticated by ATCC

Mycoplasma contamination

All cell lines tested negative for mycoplasma contamination

Commonly misidentified lines
(See ICLAC register)

n/a

Plants

Seed stocks

Report on the source of all seed stocks or other plant material used. If applicable, state the seed stock centre and catalogue number. If plant specimens were collected from the field, describe the collection location, date and sampling procedures.

Novel plant genotypes

Describe the methods by which all novel plant genotypes were produced. This includes those generated by transgenic approaches, gene editing, chemical/radiation-based mutagenesis and hybridization. For transgenic lines, describe the transformation method, the number of independent lines analyzed and the generation upon which experiments were performed. For gene-edited lines, describe the editor used, the endogenous sequence targeted for editing, the targeting guide RNA sequence (if applicable) and how the editor was applied.

Authentication

Describe any authentication procedures for each seed stock used or novel genotype generated. Describe any experiments used to assess the effect of a mutation and, where applicable, how potential secondary effects (e.g. second site T-DNA insertions, mosiacism, off-target gene editing) were examined.

Flow Cytometry

Plots

Confirm that:

- [x] The axis labels state the marker and fluorochrome used (e.g. CD4-FITC).
- [x] The axis scales are clearly visible. Include numbers along axes only for bottom left plot of group (a 'group' is an analysis of identical markers).
- [x] All plots are contour plots with outliers or pseudocolor plots.
- [x] A numerical value for number of cells or percentage (with statistics) is provided.

Methodology

Sample preparation	Transfected cells were detached by trypsin, counted and combined to 1x10^6 cells in FACS buffer, cells were stained as per methods, before being passed through a 100um filter and loaded into the cytometer.
Instrument	Accuri C6 Plus Flow Cytometer (BD Biosciences).
Software	FlowJo was used for analysis
Cell population abundance	Small fractions of cells were acquired to confirm their purity. Purity was 90-95% in our gating strategy, this was followed by acquisition of 50,000 cells per sample
Gating strategy	Cells were first gated for singlets by forward and side scatter, then live/dead stain and PE to determine cells positive for HA tag without membrane permeabilization, indicating HA-DUOX2 plasma membrane localization. Non stained and secondary antibody only stained samples were used as controls to determine the boundaries for HA-DUOX2 positive and negative cells. A live/dead control was used to indicate live cell populations.

- [ ] Tick this box to confirm that a figure exemplifying the gating strategy is provided in the Supplementary Information.
